# Editorial: NLRP3 Inflammasome: Regulatory Mechanisms, Role in Health and Disease and Therapeutic Potential

**DOI:** 10.3389/fimmu.2021.765199

**Published:** 2021-09-20

**Authors:** Oleg V. Chernikov, Jong-Seok Moon, Ann Chen, Kuo-Feng Hua

**Affiliations:** ^1^G.B. Elyakov Pacific Institute of Bioorganic Chemistry, Far Eastern Branch, Russian Academy of Sciences, Vladivostok, Russia; ^2^Department of Integrated Biomedical Science, Soonchunhyang Institute of Medi-bio Science (SIMS), Soonchunhyang University, Cheonan, South Korea; ^3^Department of Pathology, Tri-Service General Hospital, National Defense Medical Center, Taipei, Taiwan; ^4^Department of Biotechnology and Animal Science, National Ilan University, Ilan, Taiwan; ^5^Department of Medical Research, China Medical University Hospital, China Medical University, Taichung, Taiwan

**Keywords:** NLRP3 inflammasome, inflammatory disease, therapeutic approach, drug target, inflammation

Inflammasomes are cytoplasmic host defence machinery whose activation is critical for initiating signalling pathways for effective innate immunity. Although there are multiple inflammasomes, the NLRP3 inflammasome has attracted the attention of the global scientific community. The ability this inflammasome to be activated by multiple stimuli provides ample opportunities to understand its role in the development of diseases. Regulating NLRP3 inflammasome activation and deactivation is equally important and can be managed at different levels. NLRP3 inflammasome activation and the associated secretion of proinflammatory cytokines are required to initiate adaptive responses against pathogens. This is particularly useful in the development of several adjuvants to formulate effective vaccines. NLRP3 inflammasome activation is critical for the maintenance and trafficking of haematopoietic stem cells and haematopoiesis. However, NLRP3 inflammasome hyperactivation and prolonged activity cause pyroptotic cell death and sterile inflammation, causing several health complications. Thus, the NLRP3 inflammasome is an interesting protein complex in our immune system, and targeting this complex will enable tremendous therapeutic advantages. Although multiple findings are being reported every day regarding the contradictory roles of the NLRP3 inflammasome in health and disease, there is still much that is not known. A deeper understanding of this comprehensive protein complex will require new strategies and methodologies, which will take us to a new era of therapeutics. Considering the rapid growth of interest in the NLRP3 inflammasome, we are delighted to present a focused issue featuring 24 specifically commissioned cutting-edge research and review articles that shed light on recent developments in the NLRP3 inflammasome.

## NLRP3 Inflammasome in Health

In certain contexts, activation of the NLRP3 inflammasome helps control disease pathogenesis. Various oncogenic stresses and metabolic abnormalities are known to activate the NLRP3 inflammasome, which determines the fate of tumours. To avoid the expansion of tumours and achieve control in metastasis, it is important to understand the smooth transition of the NLRP3 inflammasome from protumorigenic to antitumorigenic functions. Lin et al. reviewed the tumour-suppressing and tumour-promoting roles of the NLRP3 inflammasome in different types of cancers in detail. Inhibiting the effects of the NLRP3 inflammasome may not be beneficial in treating certain cancers (Lin et al.). Tezcan et al. reported that inhibiting the activity of caspase-1 using VX765 promoted tumour growth by enhancing angiogenesis and releasing the cytokines C-C Motif Chemokine Ligand (CCL) 26, CCL11 and CCL24. VX765 also preserved the tumour cell viability. The authors showed that increasing the levels of IL-1β and IL-18 by activating the NLRP3 inflammasome using nigericin was an effective therapeutic approach to control tumour growth. Hence, for effective personalized cancer treatments, it is important to test the prior effect of NLRP3 inflammasome activation on tumours (Tezcan et al.). Such interesting insights undoubtedly assist in developing novel therapeutic strategies to treat various forms of cancers.

A review by Ratajczak and Kucia discussed the role of extracellular ATP (eATP) and extracellular ADP (eADP) in controlling NLRP3 inflammasome activation in haematopoietic progenitor stem cell (HPSC) trafficking in depth. Although eATP and eADP are purinergic mediators, eATP has been shown to activate the NLRP3 inflammasome, while eADP inhibits activation. HPSCs activate NOX-2 in response to chemoattractants from bone marrow. NOX-2 acts as an ROS source to activate the NLRP3 inflammasome, which releases ATP extracellularly. eATP further activates the NLRP3 inflammasome in a feedback manner. CD39 and CD73 are present on the cell surface and convert eATP to eADP, further activating haem oxygenase-1 through P1 receptors. This effect of eADP negatively regulates the activation of the NLRP3 inflammasome. HPSC homing from bone marrow to peripheral blood and vice versa is a process that is critically dependent on the activity of the NLRP3 inflammasome (Ratajczak and Kucia).

Currently, the major treatment for diseases involves immunosuppression by inhibiting inflammasome or interleukin (IL)-1β activity. However, this treatment usually paves the way for additional respiratory tract infections because of immunosuppression. Surabhi et al. reviewed the beneficial effect of NLRP3 inflammasome activation against microbial infections. IL-1β plays an important role in antagonizing the invasion of bacteria. Achieving immunosuppression with inhibitors of inflammasomes or IL-1β negatively affects patient health by exposing patients to invasive pneumococcal infections that can cause severe pneumonia. Deeper insights into the role of the NLRP3 inflammasome in such bacterial infections and the effect of inflammasome inhibition would help us to develop therapeutics that could significantly improve the health of immunocompromised individuals (Surabhi et al.).

## NLRP3 Inflammasome in Diseases and Therapeutic Strategies

Identifying novel therapeutic strategies is crucial in preventing unwanted activation of the NLRP3 inflammasome. Lin et al. showed that reprogramming pyruvate metabolism could be a potential therapeutic tactic to suppress the activation of the NLRP3 inflammasome. The authors showed that decreasing the activity of pyruvate dehydrogenase E1 subunit α1 and mitochondrial pyruvate carrier 2 deceased pyruvate oxidation and enhanced the activation of the NLRP3 inflammasome. An *in vivo* study carried out in a mouse model showed that peritonitis decreased with the inhibition of lactate dehydrogenase. This study reported that lactic acid fermentation was crucial for the activity of the NLRP3 inflammasome, whereas the conventional pyruvate oxidation pathway was not (Lin et al.).

Chang et al. investigated the negative regulation of the NLRP3 inflammasome by insulin in detail. Insulin significantly reduced inflammation by preventing inflammasome assembly by inhibiting ASC speck formation and IL-1β secretion and altering the phosphorylation of p38 MAPK and Syk. *In vivo* studies showed that insulin supplementation in LPS-exposed mice reduced the inflammation associated with intestinal injuries (Chang et al.). Lin et al. showed that gallic acid improved gouty arthritis by inhibiting LDH release and ROS generation. Gallic acid also reduced macrophage and neutrophil migration in joint synovitis. Gallic acid prevented pyroptosis by blocking NLRP3 inflammasome assembly, ASC oligomerization, NLRP3-NEK interactions, caspase-1 activation, and IL-1β release. Furthermore, gallic acid induced the antioxidant pathway by enhancing the expression of Nrf-2 (Lin et al.). Wu et al. identified that analogues of the polyenyl pyrrole derivative 4-hydroxy auxarconjugatin B (F240B) inhibited the NLRP3 inflammasome through autophagy induction. Apart from enhancing autophagic flux, LC3 puncta, and acidic vesicular organelle formation, F240B inhibited pro-IL-1β expression, ASC speck formation, oligomerization, and mitochondrial membrane integrity loss and promoted the degradation of NLRP3 and IL-1β. *In vivo* studies in an MSU-induced peritonitis model showed that F240B reduced the levels of IL-6, IL-1β, caspase-1, and MCP-1 and intraperitoneal neutrophilic flux (Wu et al.).

Long noncoding RNAs (lncRNAs) play significant roles in regulating the activation of the NLRP3 inflammasome. The nuclear and cytoplasmic regulation of the NLRP3 inflammasome by lncRNAs occurs by altering gene transcription, translation, and chromatin architecture. Menon and Hua published a consolidated review on various aspects of lncRNA-based regulation of the NLRP3 inflammasome and potential therapeutic candidates targeting these regulatory lncRNAs (Menon and Hua). Seok et al. presented a highly focused review on NLRP3 inflammasome regulatory activities post translation and by small molecule inhibitors and phytochemicals. Multiple intracellular proteins are responsible for the positive and negative posttranslational regulation of the NLRP3 inflammasome. These proteins carry out actions such as phosphorylation, dephosphorylation, ubiquitination, deubiquitination, sumoylation, and desumoylation to control the activity of the NLRP3 inflammasome. Similarly, numerous synthetic small molecules block the activity of the NLRP3 inflammasome by targeting mainly the NLRP3-NATCH domain, caspase-1, and IKKβ kinase activity. Phytochemicals, on the other hand, target the NLRP3-PYD domain and ASC-PYD domain to inhibit the interaction of NLRP3 with inflammasome components, ROS generation, and ASC speck formation (Seok et al.).

Suryavanshi et al. presented a detailed review on the promising therapeutic potential of cannabinoids, an active component of cannabis from the plant *Cannabis sativa*, against the activation of the NLRP3 inflammasome. The potential anti-inflammatory activity of cannabinoids can be utilized to formulate therapeutics targeting various inflammatory conditions, especially COVID-19-induced acute respiratory distress syndrome (Suryavanshi et al.). There is increasing interest in uncovering the regulatory effects of various nuclear receptors on the NLRP3 inflammasome. Alatshan and Benko reviewed the significant role of nuclear receptors in modulating the activity of the NLRP3 inflammasome in detail. Ligand-mediated interactions of nuclear receptors inhibit the activation of the NLRP3 inflammasome. Such integration interferes with and inhibits NLRP3 inflammasome assembly and ASC oligomerization, prevents ER stress, and reduces ERK activation. However, certain nuclear receptor-ligand interactions result in the activation of the NLRP3 inflammasome, which can be directed to boost immune responses in the host (Alatshan and Benko). Pyrillou et al. provide an up-to-date review of the biology of IL-1, their production, and their significant roles in multiple cell types. IL-1 play significant roles in thrombosis by promoting hypercoagulation, promoting fibrosis, delaying wound healing, driving cytokine storms and causing sepsis. A better understanding of the cell-dependent functions of IL-1 will undoubtedly help in formulating safe therapeutics with maximum efficacy to improve the quality of human health (Pyrillou et al.). Using bioinformatics tools, computational modelling and assessments, Samson et al. determined an inverse correlation between NLRP3 mutation-associated structural changes and disease severity. One of the key findings reported was that severe structurally interfering NLRP3 mutations localize around the ATP binding domain, altering the charge interactions and hydrogen bonding, thereby enhancing the ATP binding affinity. Moderately interfering structural NLRP3 mutations enhance NLRP3 inflammasome formation and stability by influencing the binding of NLRP3-NLRP3 multimers and NLRP3-ASC proteins (Samson et al.).

Dong et al. illustrated the importance of peptidyl-prolyl cis-trans isomerase (Pin1) in regulating the activation of the NLRP3 inflammasome to reduce LPS-induced septic shock in mice. *In vivo* studies in the absence of Pin1 showed reduced activation of the NLRP3 inflammasome. Pin1 positively regulated the phosphorylation of p38 MAPK and affected the expression of NLRP3, ASC, and caspase 1. Pin1 is also important for the cleavage of gasdermin D and enhances the release of the inflammatory cytokines IL-1β and IL-18 (Dong et al.). Lan et al. demonstrated the importance of the PKR/peroxynitrite axis in initiating inflammasome activation in LPS-challenged cardiac fibroblasts. LPS exposure enhanced the phosphorylation of PKR, which in turn mediated the expression of pro-IL-1β. Using inhibitors, the authors demonstrated that PKR inhibition before LPS priming reduced the levels of NLRP3 and pro-IL-1β, while inhibiting these factors after LPS priming but prior to activation decreased the activation of caspase-1 and IL-1β. Inhibiting peroxynitrite negatively affected the priming and activation of the NLRP3 inflammasome. PKR/peroxynitrite can be considered a potential therapeutic target to suppress NLRP3 inflammasome-mediated inflammatory responses in septic shock (Lan et al.).

A manuscript by Sakaguchi et al. showed that deficiency of the autophagy-related proteins Gate-16 and GABARAP enhanced the activation of guanylate binding protein 2 associated caspase-11, which directly increased the inflammatory responses usually observed in LPS-induced septic shock. Critical crosstalk and interregulation between the NLRP3 inflammasome and autophagy are crucial in preventing hyperinflammation. A review by Biasizzo and Kopitar-Jerala discussed the inhibition of NLRP3 inflammasome hyperactivation by the induction of autophagy. Autophagy inhibits NLRP3 inflammasome activation by removing NLRP3-activating triggers, activators, cytokines and inflammasome components. NLRP3 inflammasome signalling events also regulate autophagy to establish an optimal balance between host defence and hyperinflammation (Biasizzo and Kopitar-Jerala).

Inhibiting NLRP3 inflammasome activation can effectively ameliorate mosquito-borne dengue virus-driven dengue haemorrhagic fever (DHF) pathogenesis. In a study conducted by Lien et al., the NLRP3 inflammasome was demonstrated to be a potential therapeutic target to overcome the platelet defects induced by dengue. Virion surface envelope protein domain III (EIII)-induced platelet defects and cell death by pyroptosis were inhibited by blocking the activity of the NLRP3 inflammasome using the inhibitors OLT1177 and Z-WHED-FMK (Lien et al.). Lien et al. showed that EIII caused the release of neutrophil extracellular traps and induced a form of cell death known as NETosis and cytokine storms, which were mainly mediated by IL-1β and IL-18 through the activation of the NLRP3 inflammasome. *In vitro* studies using Z-WHED-FMK and OLT1177 showed the suppression of NETosis induced by EIII. Furthermore, *in vivo* studies with mutant mice lacking *Nlrp3* and *Casp1* also showed reduced NETosis and inflammation after EIII exposure (Lien et al.). The vascular endothelial dysfunction observed in cases of DHF is caused by enhanced pyroptotic death in endothelial cells. By inhibiting the activation of the NLRP3 inflammasome and caspase 1, the inflammatory pathogenesis associated with DHF can be significantly reduced (Lien et al.). Thus, the NLRP3 inflammasome is a potential therapeutic target in dengue infections.

Deng et al. analysed the role of the lncRNA Gm28309 in NLRP3 inflammasome activation during brucellosis. The lncRNA Gm28309 acts as a competitive endogenous RNA to sponge the NF-κB-activating activity of miR30685p. This ensures the suppression of unwanted NLRP3 inflammasome activation under normal conditions. However, during *Brucella* infection, TLR activation results in the downregulation of the lncRNA Gm28309, which releases miR30685p to the cytoplasm, where it targets κB-Ras2 for degradation and results in the phosphorylated activation of p65. This phosphorylation activates the NF-κB signalling cascade, which culminates in the activation of the NLRP3 inflammasome and IL-1β release to mediate inflammatory activity. Downregulation of the lncRNA Gm28309 also increases the expression of TGF-β, which leads to the phosphorylation of p65 by activating the TAK1 and IKK kinase cascades (Deng et al.).

Song et al. reported that therapeutically targeting NLRP3 inflammasome activation in Shiga toxin-induced haemolytic uraemic syndrome could effectively ameliorate inflammatory pathogenesis. The small-molecule inhibitor oridonin effectively suppressed NLRP3 inflammasome activation and improved survival in an interleukin mouse model by preventing renal injuries (Song et al.). The deadliest respiratory infection that the world is battling today is SARS-CoV-2-induced acute respiratory distress syndrome. COVID-19 induces hyperactivation of inflammatory responses and enhances the production of IL-1β and IL-6. Vaccari et al. NLRP3 inflammasome activation by COVID-19 and its role in triggering disseminated intravascular coagulation, ARDS, and ventilator-induced lung injury in detail. The authors also explained in detail the detrimental effect of NLRP3 inflammasome-induced responses in various organs during SARS-CoV-2 infection. Therapeutics that inhibit NLRP3 inflammasome activation and IL-1β and IL-6 signalling could provide extensive benefits to SARS-CoV-2-infected individuals (Vaccari et al.).

NLRP3 inflammasome activation is regulated at various levels. It is critical to understand the multiple regulatory events involved in controlling the activity of the NLRP3 inflammasome. With the rising incidences of inflammatory diseases, deeper knowledge at the molecular level will enable the development of targeted drug formulations with greater efficacy.

## Author Contributions

K-FH is the guarantor of the article. OC, J-SM, and AC contributed to critical revision of the manuscript. K-FH wrote and finished the manuscript. All authors contributed to the article and approved the submitted version.

## Funding

This research work is supported by the funding from the Ministry of Science and Technology of Taiwan (MOST 110-2628-B-197-001; MOST 108-2923-B-197-001-MY3; MOST 110-2923-B-197-001-MY3).

## Conflict of Interest

The authors declare that the research was conducted in the absence of any commercial or financial relationships that could be construed as a potential conflict of interest.

## Publisher’s Note

All claims expressed in this article are solely those of the authors and do not necessarily represent those of their affiliated organizations, or those of the publisher, the editors and the reviewers. Any product that may be evaluated in this article, or claim that may be made by its manufacturer, is not guaranteed or endorsed by the publisher.

